# Acute lead intoxication in a female battery worker: Diagnosis and management

**DOI:** 10.1186/1745-6673-5-19

**Published:** 2010-07-07

**Authors:** George Dounias, George Rachiotis, Christos Hadjichristodoulou

**Affiliations:** 1 Department of Occupational & Industrial Hygiene National School of Public Health, Athens, Greece; 2 Department of Hygiene and Epidemiology, Medical Faculty, University of Thessaly, Larissa, Greece

## Abstract

Lead is a significant occupational and environmental hazard. Battery industry is one of the settings related to lead intoxication. Published information on the use of oral chelating agents for the treatment of anaemia in the context of acute lead intoxication is limited. The patient was a 33 year immigrant female worker in a battery manufacture for 3 months. She complained for malaise that has been developed over the past two weeks. Pallor of skin and conjunctiva was the only sign found in physical examination. The blood test on admission revealed normochromic anaemia. Endoscopic investigation of the gastrointestinal system was negative for bleeding. The bone marrow biopsy was unrevealing.

At baseline no attention has been paid to patient's occupational history. Afterwards the patient's occupational history has been re-evaluated and she has been screened for lead intoxication. The increased levels of the lead related biomarkers of exposure and effect confirmed the diagnosis. The patient received an oral chelating agent and an improvement in clinical picture, and levels of haematological and lead related biochemical parameters have been recorded. No side effect and no rebound effect were observed. This case report emphasizes the importance of the occupational history in the context of the differential diagnosis. Moreover, this report indicates that lead remains a significant occupational hazard especially in the small scale battery industry

## Background

Lead is a significant occupational and environmental hazard. Lead poisoning has been identified as an occupational hazard from ancient times. This was the case with Hippocrates. During the modern era lead intoxication has been studied by the pioneers of occupational medicine Bernardino Ramazzini, and Alice Hamilton [1]. Battery industry is one of the settings related to lead intoxication [2-4]. Despite the fact that lead toxicity has been widely studied, however nowadays there are significant gaps related to the management of exposure to lead. A notable gap is related to lead chelation given that it is performed empirically, and no consensus guidelines do exist and controlled clinical trials are not available to confirm the various biological effects of chelation [5-7].

In Greece 6% of the recognized occupational morbidity has been attributed to lead exposure, however, it is anticipated that this figures represent an underestimation of the true lead- related occupational morbidity [8].

Succimer (2, 3 dimercaptosurinic acid) is an orally administered chelating agent which recently has been increasingly used. There is some evidence that Succimer is better tolerated and has a wider therapeutic index than dimercaprol and it has been considered as less toxic than other chelating agents [7].

Published information on the use of oral chelating agents for the treatment of anemia in the context of acute lead intoxication is limited [9,10].

In this case report we present a case of a patient suffering from acute occupational lead poisoning occupationally exposed to lead who underwent successful chelation by the use of an oral chelating agent.

## Case presentation

The patient was a 33 year immigrant female worker in a battery factory for 3 months. The women worked as a cleaner in the plan. The main activity of the factory was to use to gain lead from old batteries. The female worker reported that the working environment was dusty, the ventilation was poor, and she didn't use respiratory protection. The worker complained for malaise that has been developed over the past two weeks. In addition she reported mild pain in the abdomen. Pallor of skin and conjuctiva was the only sign found in physical examination. Her temperature was normal, with a respiratory rate of 16/min. The Electrocardiogram was normal and the pulse rate: 78/min. The blood test on admission revealed normochromic anemia, in addition haemolysis was present. In particular, the results were as follows: Hematocrit: 23.8% (34-46); Hemoglobin: 8 gr/dl (12-16); Platelet Count: 197.000(150000-400000); White blood cells count: 4.900 μL (mm^3^) (500-11000); Red blood cells count: 2.66 μL(mm^3^) (4.0-5.2); Mean corpuscular volume: 88 (70-100); Mean cell hemoglobin: 29.5 pg (26-34); Mean cell hemoglobin concentration: 32.5% (31-37); Reticulocyte: 4.9% (0.5-1.5). However, bilirubin, and lactate dehydrogenase were within the normal range. The peripheral smear reveled constant basophilic stippling. Regarding biochemical parameters blood sugar serum creatinine, and liver function enzymes were within normal limits. Endoscopic investigation of the gastrointestinal system was negative for bleeding. The bone marrow biopsy was unrevealing. At baseline no attention has been paid to patient's occupational history.

Given the negative results of the above mentioned medical tests a detailed occupational history has been obtained. On the basis of the occupational history the patient has been screened for lead intoxication. After admission to the hospital the patient has been removed from exposure. The laboratory methods used for the measurement of blood lead and ZZP concentrations were Atomic Absorption Spectrometry, and Hematofluorometric method, respectively.

The patient had high blood levels:90 μg/dl. Increased levels have been also been observed for other markers of lead intoxication like δ-aminolaevulinic acid (urine). On the contrary the levels of Zinc Protoporphyrin (ZZP) were normal (Table 1). The diagnosis of acute lead intoxication has been established, and the patient has received a 19 day course of the oral chelating agent succimer (Chemet). The dosage was as follows: 30 mg/Kg body weight every eight hours for the first dive days and 30 mg/Kg body weight every twelve hours for 15 days. The patient has been adequately hydrated. In addition complete blood count with white blood cell differential and platelet counts were obtained weekly during chelation therapy. Renal, liver function and electrolyte levels were also monitored weekly. We didn't observe any notable variation regarding renal, liver function or electrolyte levels.

**Table 1:** Levels of hematological parameters before and after oral chelation

Parameter	On admission	On discharge	One month after chelation
Hemocrit	23.8	26.5	34.7

Hemoglobin (g/dl)	8.0	8.6	12.1

Reticulocyte Count	4.9	2.1	1.5

Platelet Count	197	220	303

After the initiation of chelation the symptoms of the patient have been ameliorated while a notable improvement of the hematological parameters has been recorded. Especially, hematocrit and hemoglobin have been gradually elevated (Table 1). Furthermore, a considerable reduction in the levels of the biological indicators of exposure (blood lead) and effect (δ-ALA, urine) have been observed (Table 2).

**Table 2:** Levels of lead related biological indices before and after oral chelation

Parameter	On admission	One month after chelation
Blood Lead (μg/100 ml)	90	17

ZZP (μg/gr creatinine)	16.5	15.5

ALA-U (mg/gr creatinine)	81.5	7.6

At the time of discharge the levels of blood lead and ZZP were 37 μg/ml, and 13 μg/gr creatinine, respectively.

The measurements of the concentrations of the blood levels have been repeated for three consecutive months, and the blood lead levels found to be < 40 μg/ml.

The patient didn't report any side-effect during the administration of the chelating agent.

## Discussion

We presented a case of acute lead intoxication in a female worker in Small Size battery factory. The factory mainly worked in gaining lead from old batteries which is a dangerous activity for the workers. The female employee worked as a cleaner and was exposed to lead dust. Her occupational exposure to lead was uncontrolled. She didn't used respiratory protection, and the ventilation in the workplace reported to be poor. In addition, no lead surveillance program has been implemented in the battery industry unit. Almost all medical tests performed for the investigation of the anemia (with hemolysis and basophilic stippling) which the patient presented were unrevealing. Initially no attention has been paid to patient's occupational history, however- after negative tests results - the occupational history of the patient was reconsidered and the lead intoxication was included in the differential diagnosis. Indeed, the level of lead related markers of exposure (blood lead), and effect (δ-ALA) was well above the standards defined by the Greek legislation [11] ("action level": ≥ 40 μg/dl and "maximum permissible level":70 μg/dl for blood lead; "maximum permissible level" for δ-ALA: < 20 mg/gr creatinine), thus the diagnosis of lead intoxication has been documented. It should be mentioned that the Greek standards for the biological monitoring of workers exposed to lead are higher in comparison to the limits included in the regulations of Occupational Safety and Health Administration (OSHA) [12]. Nevertheless, it should be underlined that data from Greece on environmental and biological monitoring of workers occupational exposed to lead are sparse.

However, on the basis of the worker's clinical data there could be the suspicion of her suffering from thalassaemia or another hematological disorder which could be unrelated to the absorption of lead. Moreover, the laboratory finding of basophilic stippling in peripheral smear could also be seen in thalassaemia as an indication of a defect in protein synthesis.

This alternative diagnosis seems unlikely given that the Mean Cell Hemoglobin (MCH) and Mean Cell Hemoglobin Concentration (MCHC) were within the normal range, and no patient's history of thalassaemia was recorded. Furthermore an elevated ZZP could also occur in thalassaemia trait; however the patient has recorded normal levels of ZZP. Finally it is of interest that the bone marrow biopsy was unrevealing and this finding does not support the presence of a hematological disorder as the cause of the anemia.

The patient received an oral chelating agent (Succimer) and an improvement in clinical picture, and levels of hematological and lead related biochemical parameters have been recorded. No side effect and no rebound effect were observed. The last is additional evidence suggesting the absence of a significant body burden of lead [13].

In conclusion, this case report emphasizes the importance of the occupational history in the context of the differential diagnosis. When physicians properly ask patients about their occupational history this could be helpful preventing patient from underwent unnecessary, costly and sometimes potentially harmful medical testing [14,15]. Moreover, the present report indicates that lead exposure could be an uncontrolled hazard especially in the small size battery industry. Finally, oral chelation- together with worker's removal from exposure to lead- was effective and safe in the management of the present case of acute lead intoxication.

## Consent

Written informed consent was obtained from the patient for publication of this case report and any accompanying images. A copy of the written consent is available for review by the Editor-in-Chief of this journal.

## Competing interests

The authors declare that they have no competing interests.

## Authors' contributions

GD contributed in visiting the case, and in revising the manuscript. GR drafted and revised the manuscript. CH revised the manuscript for important intellectual content. All authors have read and approved the manuscript.
